# From air to mind: unraveling the impact of indoor pollutants on psychiatric disorders

**DOI:** 10.3389/fpsyt.2024.1511475

**Published:** 2025-01-09

**Authors:** German Torres, Ryia T. Subbaiah, Riya A. Sood, Joerg R. Leheste

**Affiliations:** ^1^ Department of Biomedical Sciences, New York Institute of Technology College of Osteopathic Medicine (NYITCOM), Old Westbury, NY, United States; ^2^ Department of Arts and Sciences, Georgetown University, Washington, DC, United States

**Keywords:** epidemiology, volatile organic compounds (VOCs), anthropogenic pollutants, psychiatric disorders, indoor air pollution, mood disorders, neural circuits, genetics

## Abstract

Epidemiological evidence from the past 20 years indicates that environmental chemicals brought into the air by the vaporization of volatile organic compounds and other anthropogenic pollutants might be involved, at least in part, in the development or progression of psychiatric disorders. This evidence comes primarily from occupational work studies in humans, with indoor occupations being the most important sources of airborne pollutants affecting neural circuits implicated in mood disorders (e.g., major depressive disorder and bipolar disorder). The current mini review brings together recent findings of indoor airborne pollution from different fields of research, including genetics, neuropathology, and neuroimaging, for gauging underlying physiological mechanisms leading to emotional disturbances that impact nearly all aspects of human behavior. A better understanding of how indoor airborne pollutants affect brain neurons to augment clinical symptoms associated with psychiatric disorders will undoubtedly be useful in the subsequent treatment of patients with major depressive and/or bipolar disorders. This article is part of the themed issue, “Understanding the Link Between Environmental Pollutants, Brain & Behavior.”

## Introduction

**Figure 1:**
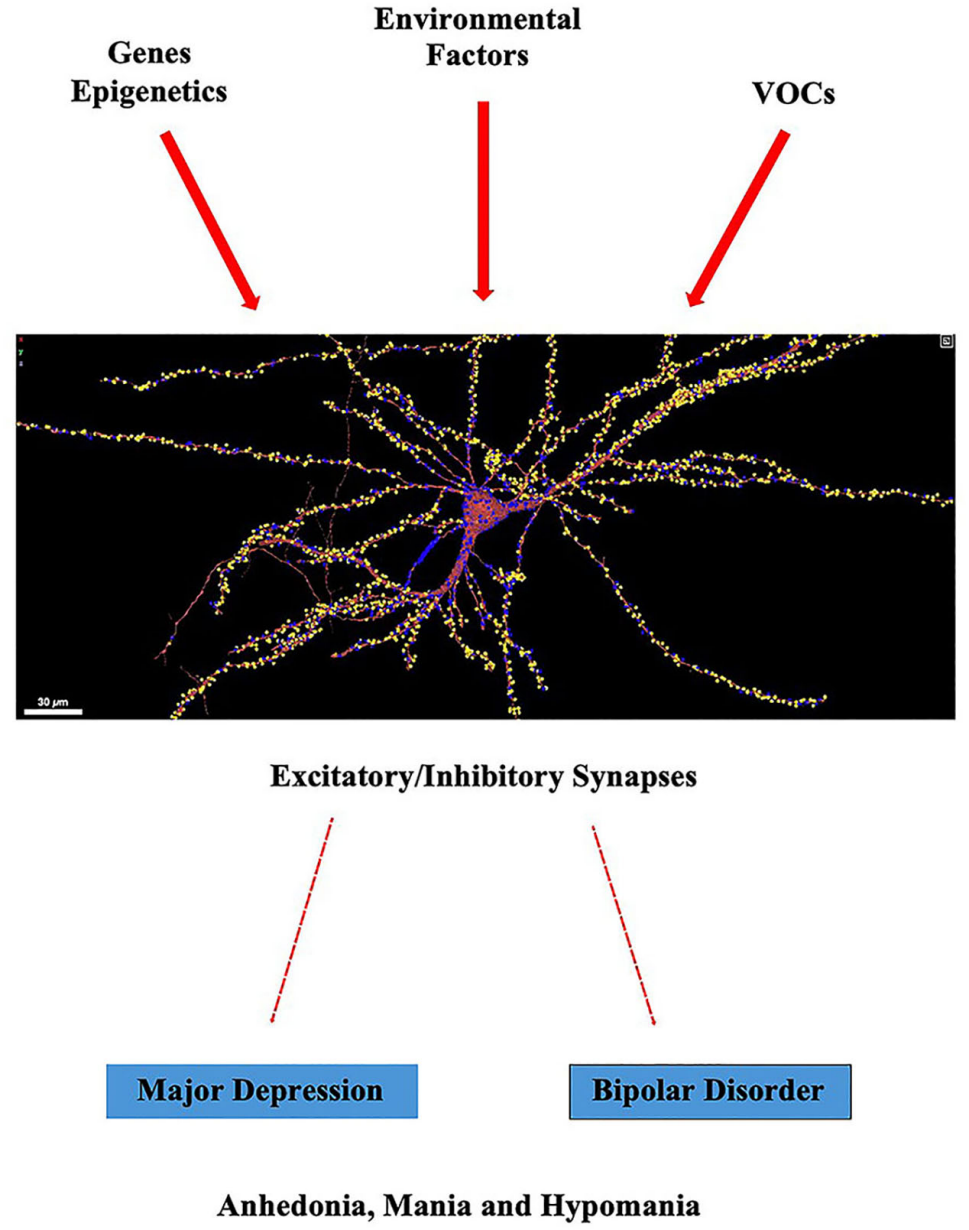
Development and progression of mood disorders (e.g., major depressive disorder and bipolar disorder) are thought to have several causative variables ranging from disruptions of gene expression programs, including epigenetic modifications, to environmental insults, potentially restricting the flow of excitatory/inhibitory chemical signals between synapses. VOCs, such as organophosphate flame retardants, may not necessarily contribute directly to DNA instability, but instead, they may increase the risk of psychiatric illnesses by indirectly affecting the functions of neurons and glia, as well as being linked to physiological illnesses such as heart and lung disease. As neurons and glia drive human behavior, including externalizing behaviors, inhalation of VOCs within closed spaces might exacerbate clinical symptoms of anhedonia, mania, and hypomania, core trait factors in mood disorders. Image of a reconstructed neuron from the human cortex using electron microscopy obtained from NeuroGlancer.

Humans are constantly exposed to a myriad of airborne pollutants that strike the mucous membranes of the eyes (e.g., the conjunctiva), nose (e.g., nasal mucosa), and mouth (e.g., oral mucosa). If the pollutants are fine particulate matter (PM, less than 2.5 micrometers in aerodynamic diameter), they are inhaled into the lungs through the tortuous airways of the trachea, bronchi, and bronchioles. Each absorbed pollutant contributes more or less subtly to a physiological state of cellular and organismal dysfunction, causing disease and worsening morbidity outcomes as evident from epidemiological and experimental data (1, 2). Volatile organic compounds (VOCs), for example, can have a negative impact on tissues and organs that make up the bilateral body plan by increasing an individual’s susceptibility to respiratory, cardiovascular, immunity, endocrine, and possibly cancer development (**Table 1**). As the brain is the skeleton for cognition, airborne pollutants may also synergistically affect neural circuits or networks that are most likely to be causal for psychiatric traits (3–6). For example, mood disorders (e.g., major depressive disorder and bipolar disorder) are psychiatric illnesses thought to be linked to multiple levels of neurobiology, including cognitive behavior, stress hormone secretion, neurotransmitter-receptor signaling, DNA methylation, innate immune function, and non-coding RNA sequences that regulate gene expression, both at the transcriptional and post-transcriptional level. Thus, deviations of large-scale functional brain networks within disease-specific genomic contexts appear to underlie the etiology and pathogenesis of mood disorders (7, 8). As airborne pollutants are ubiquitous throughout urban and regional environments (9, 10), they are thought to exacerbate the neural network disorder that generates obtrusive clinical symptoms of anhedonia, mania, and hypomania often diagnosed in patients with major depressive and bipolar disorders, respectively (**Figure 1**).

**Table 1:** Adverse Health Outcomes Following Exposure to VOCs. Anthropogenic release of VOCs worsens air quality, thus resulting in sustained inflammation, oxidative stress, and increased risk of disease.

Cellular/Organismal Effects	Health Risks	References
Generation of Reactive Oxygen Species	Inflammation and cellular damage	(97)
Respiratory Disease	Asthma and bronchitis	(97, 98)
Cardiovascular Disease	Heart disease and hypertension	(98, 99)
Neurological Disorder	Neurotoxicity; Reduced cognitive function	(97)
Carcinogenic Disease	Increased risk of cancer (directly)	(97)
Endocrine Disruption	Hormone dysregulation; Reduced reproductive status	(100)
Immune Dysfunction	Reduced immune fitness; infections; cancer (indirectly)	(100)

Humans are exposed to ubiquitous VOCs that can impact different groups of cells to cause disease *via* direct or indirect biological mechanisms. VOCs have both acute and chronic effects on human health, affecting a number of different systems and organs, including the brain.

It should be noted that humans are bombarded daily with airborne pollutants originating from various geographical sources: from above, as satellites and spacecraft reentry the earth’s atmosphere, they combust and release toxic metal particles (e.g., aluminum, AL; copper, CU) and noxious gasses (e.g., hydrogen sulfide, HS_2_), bringing down with them significant health risks for humans and their ecosystems (11, 12). Anthropogenic pollutants from the soil (e.g., nanoplastics) and groundwater (e.g., polyvinyl chloride, PVC polymers) also increase the risk of disease susceptibility as their average airborne concentrations (typically measured in parts per billion or milligrams per cubic meter, mg/m^3^; < 15 mg/m^3^ permissible exposure limits) often exceed the WHO-recommended threshold values for highly urbanized areas (13, 14). However, a more intimate or proximate source of airborne pollution for humans is their own familial and social habitats, such as homes and public buildings (15). Here, the spatial distribution of pollutants, magnitude of pollution, and temporal exposure to pollutants are amplified severalfold, as closed spaces, long-term occupancy, poor indoor ventilation, and proximity to others in particular have a deleterious impact on several health outcomes, including pulmonary and cardiovascular illness and mild cognitive impairment. To expand this important clinical theme a bit further, we carried out a systematic PubMed literature review on indoor exposure to airborne pollutants and their potential association with structural and functional changes of neural networks specialized in emotional and cognitive behavioral underpinnings. There are five main areas covered in this perspective, including: I) nature of indoor airborne pollutants and toxicity mechanisms; II) oxidative stress, inflammation, and activation of neural circuits; III) impact of indoor airborne pollutants on the epidemiology of mood disorders; IV) social health markers, emotional cognition, and indoor airborne pollution; and V) appropriate measures for reducing the adverse effects of indoor airborne pollutants on brain-signaling pathways.

## Nature of indoor airborne pollutants and toxicity mechanisms

Archaic and modern humans have been exposed to particle pollution ever since they began to inhabit coastal cave dwellings and then steel-framed buildings over the past 250,000 to 170,000 years of hominin dispersal (16, 17). The human dependence on indoor dwellings may have shaped the evolution of cognitive, social, and technological developments, but they have also brought their occupants in close proximity to carbon monoxide (CO) emissions from fire pit hearths and gas degradation byproducts (e.g., hydrogen fluoride, HF; ethylene, C_2_H_4_) from lithium-ion batteries, now commonly used in office buildings for photovoltaic energy (18, 19). Human physiology and demographic histories therefore hold a record of pollution exposure that has gradually accumulated over timescales of urban expansion. Advances in *in situ* data and more recently emerging technologies such as satellite data and mathematical algorithms have deepened our understanding of how particle pollution affects human cells of diverse types (20). These advances have also revealed that multiple indoor pollutants not only readily gain access to the brain parenchyma but also impact measurable neural correlates that can be linearly mapped onto neural networks associated with mood disorders (21).

**Figure 2:**
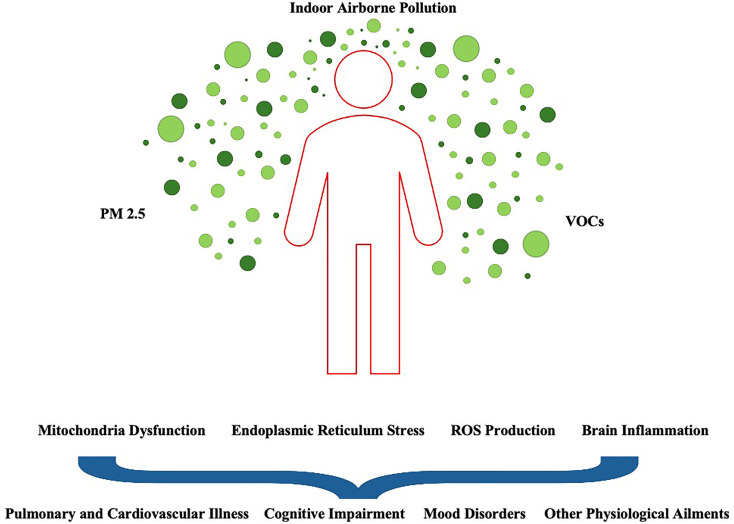
A diverse array of indoor airborne pollutants affect biological systems from cell circuits to population networks in intricate ways. Certain brain regions and their cell types may be particularly vulnerable to PM _2.5_, as their nuclear architecture, gene expression programs and chromatin states vary widely. In this context, as airborne pollutants passively flow throughout urban and regional environments, they are thought to alter the internal microenvironment that sculpts the tightly regulated and compartmentalized networks of human cells of different types. Disruption of organelle physiology (e.g., mitochondrial protein states) as a result of continuous PM_2.5_ exposure may lead to diverse clinical manifestations, including disturbances in cognitive functioning and the risk of respiratory diseases and other adverse health outcomes (e.g., metabolic syndrome).

As mentioned earlier, exposure to toxic or noxious chemicals is not restricted to specific human tissues or organs but instead presents a systemic threat to the entire bilateral body plan (22–24). VOCs such as benzene, toluene, xylenes, and formaldehyde, for instance, appear to destabilize cell membrane structures (e.g., pore-forming proteins), increase the metabolic reaction of free radicals within cells, and in some cases, induce irrevocable nuclear and cytoplasmic DNA damage to vulnerable cell clusters (25–27). Thus, all differentiated and mature cells, including nerve cells of the nervous system, share common molecular mechanisms in response to toxic-causing VOCs. It should be noted that if excessive toxicity is registered in these neurons, specific cell death-signaling pathways like autophagy (self-eating) and apoptosis (self-killing) are activated in order to regulate the fate of damaged cells within a particular functional neural network (28). Thus, there are several pathophysiological mechanisms of cytotoxicity in which an increasing number of molecules are involved (e.g., PINK1 and ERLINs), all of whom are strongly dependent on calcium (CA^2+^) flow and CA^2+^ ion concentration (29–31). Taken together, VOCs and other anthropogenic pollutants either directly or indirectly negatively affect mitochondrial function, cause endoplasmic reticulum stress, and/or influence the formation of reactive oxygen species (ROS), ultimately leading to changes in neuron- and metabolic glia-specific transcriptional programs across brain regions, behavioral states, and disease-prone populations (32, 33; **Figure 2**).

## Oxidative stress, inflammation and activation of neural circuits

Although we have made significant strides in understanding how particle pollution might detrimentally affect individual neurons and glial cells (34), it’s still not clear how indoor VOCs lead to poor health outcomes in the short-term, like irritability, or in the long-term, like endogenous depression. Nevertheless, the importance of linking noxious chemicals with ROS as a mechanism of oxidative stress is now recognized as a contributing factor in various forms of pathophysiology (35). Briefly, oxidative stress is the aberrant accumulation of ROS levels through direct or indirect aerobic metabolism, gradually weakening mitochondrial function and steady-state redox signaling. These abnormalities can lead to ion-transport deficits, cell degradative pathways, and especially neuro-inflammation, a condition characterized by innate immune activation of tissue-resident microglia (i.e., macrophages) and astrocytes in the nervous and enteric nervous systems (36, 37). Additionally, certain physiological (e.g., blood-brain barrier permeabilization) and environmental (e.g., stress hormone secretion) insults can also activate soluble cytokines such as interleukin-1B and tumor necrosis factor (e.g., TNF-alpha) within and surrounding neural networks of the prefrontal cortex, hippocampus, and amygdala (38, 39). The prefrontal cortex, hippocampus, and amygdala are brain structures specialized in cognitive function, memory consolidation, and emotional processing, respectively (40–42). Critically, a wide range of psychiatric ailments, including mood disorders, are associated with deficits of the aforementioned brain structures, potentially placing their native neurons (e.g., spiny glutamate excitatory neurons and smooth GABA inhibitory interneurons) at the threshold between VOCs exposure and the worsening of obtrusive clinical symptoms of anhedonia, mania, and hypomania (43, 44).

The fact that inflammatory processes have been implicated in the pathophysiology of mood disorders and that exposure to VOCs can potentially exacerbate innate immune responses suggests a complex interaction between activation of neural circuits and cytokine production (45–47). Thus, many signaling pathways that are linked to mood disorders are most likely affected by PM_2.5_ exposure through direct or indirect mechanisms. In general, it’s thought that major depressive disorder and bipolar disorder are caused by circuit-specific impairments of neurotransmitter-receptor signaling (48–51). For instance, deficits in electrical signal transduction from one neuron to another, faulty release of chemical neurotransmitters into the synapse, or genomic instability of cell membrane receptors might result in the breakdown of structure, organization, and function of the brain microenvironment with significant implications for psychiatric disorders. Our interpretation of these data is that indoor air pollution, through activation of a chronic neuro-inflammatory response, alters brain chemistry, notably neurotransmitter function (see below), thereby indirectly contributing to individual symptoms of anxiety, misplaced euphoria, loss of motivation, and, in extreme circumstances, suicidal ideation.

Findings from clinical and post-mortem studies of the human brain indicate a biological basis for the development of mood disorders. As mentioned earlier, neurochemistry abnormalities and state-dependent stressors (e.g., early-life adversity) have been implicated in the pathophysiology of major depressive disorder and bipolar disorder (52). For instance, deficiencies of certain monoamine neurotransmitters (e.g., 5-HT, norepinephrine, and dopamine) appear to increase the risk for the development of behavioral pathology in psychiatric conditions. However, recent studies tend to minimize the involvement of these aromatic amino acid molecules in vulnerability conditions such as anhedonia, mania, and hypomania (53). Instead, it is now thought that aberrations in the signaling integrity of excitatory glutamate neurons and inhibitory GABA interneurons lead to neuropsychiatric disease (54, 55). Regardless of which neurotransmitters are critical to nervous system dysfunction, cooking with biomass fuels (e.g., firewood) appears to deplete platelet 5-HT content and increase the prevalence of endogenous depression in women of childbearing age (56). These findings are indirectly supported by animal studies showing impairment of monoamine transmission, abnormal increases in extracellular glutamate levels, and overproduction of proinflammatory cytokines such as TNF-alpha and interleukin-1-alpha in the brains of mice exposed to VOCs and CO (57, 58). Taken together, these data suggest that indoor exposure to PM_2.5_ may directly impact the chemical activity of neurotransmitters and G protein-coupled receptors specific to the underlying pathology of mood disorders. To identify and link more toxicologically homogeneous VOCs to brain regions with neurotransmitter abnormalities, future studies will require a combination of neuroimaging approaches.

Another variable that needs to be considered in indoor airborne pollution and adverse health outcomes is gender-dependent differences. More specifically, is exposure to PM_2.5_ differently associated with chemical neuroanatomy dysfunction in men and women? Although there is evidence that household air pollutants affect women’s health more broadly than men’s, particularly in low-income settings (59), little is known about whether such gender-dependent differences extend to monoamine-, glutamate-, or GABA-signaling dysfunction. Regardless, exposure to PM_2.5_ is known to increase health risks in pregnant and nonpregnant women living in US cities with high air pollution levels. For example, a strong association between PM_2.5_ exposure and altered plasma levels of proinflammatory cytokines (e.g., interleukin-1 RA, receptor antagonist, and interleukin-18) is detected during early pregnancy (~ 20 weeks of gestation) with the possibility of long-lasting physiological and social behavioral impacts for the human offspring (60). Against this background, we argue that although there are gender-dependent differences in monoamine transmission and hormone secretion in the human brain, differences in response to PM_2.5_ exposure may simply be related to the fact that women tend to spend more time indoors than men in the context of childrearing, palliative care, and other forms of social interactions. Future studies are needed to determine whether household air pollutants such as polycyclic aromatic hydrocarbons (PAHs) normally produced from combustion of solid fuels or natural sources of ionizing radiation (e.g., radon gas, Rn) affect the brains of men and women differently, especially in terms of cognition, emotion, and behavior output.

## Impact of indoor airborne pollutants on the epidemiology of mood disorders

Improved epidemiological techniques and refined computational bioinformatic methods show that mood disorders are associated with the highest deleterious burden of any mental illness in young people aged 15–25 years (61). The growing consensus that harmful particles emanating from household items such as stoves, space heaters, or lighting devices produce subtle yet widespread structural and functional alterations in the developing brain suggests that PM_25_ might be exacerbating disease progression in certain high-risk populations. In this context, it’s thought that even prior to clinical diagnosis, young adults predicted to develop mood disorders already show increased expression of genes related to cell death and inflammation (47, 62). Thus, a genetic predisposition to emotional disturbances that converges on neural developmental programs across generations might be imposing flaws in the cell-to-cell propagation of synaptic events between local populations of excitatory glutamate and inhibitory GABA neurons (63, 64). In general, these data provide clues about the hereditary mechanisms influencing cognitive and emotional health and reveal the scale and complexity of the past and current contributions of PM_25_ to brain dysfunction in the modern human lineage.

Although the soil, groundwater, and air around us are brimming with particle pollution, they do not affect all individuals in the same way. Similarly, mood disorders do not affect all individuals in the same way, likely due to inter-individual differences in both protein-coding genes and non-protein-coding RNAs. In this context, most genetic variants linked to psychiatric disorders are located in non-protein-coding regions of the human genome, thus suggesting that brain-specific *cis*-regulatory elements (e.g., promoters and enhancers) play a significant role in the function of neurons and glial cells (65–67). It is not known, however, whether PM_25_ can infiltrate and directly interact with gene *cis*-regulatory elements to affect transcriptional programs in the interconnected neural circuits of the prefrontal cortex, hippocampus, or amygdala. In general, with ever-increasing prevalence rates of mood disorders among young adults, it’s tempting to speculate that exposure to noxious chemicals within the confined spaces of homes and offices might be accelerating the prevalence estimates for psychiatric disorders connected to a high burden of illness and poor treatment response (68). Obviously, there are other possibilities that could explain this phenomenon. For instance, one could argue that depressive-detection measures such as clinical screening and hormonal profiling have improved diagnosis and therefore have become more effective. However, most current data tend to suggest that mood disorders are not better diagnosed, but instead, they’re more clinically and symptomatically apparent in the general population (69, 70).

## Social health markers, emotional cognition and indoor airborne pollution

So far we have provided clues about the biological mechanisms by which indoor chemicals might influence cognitive and emotional health in patients who have met the diagnostic criteria for mood disorders. However, noxious chemicals are only one of several environmental risk factors that can affect brain health and wellbeing. For instance, young adults with a predisposition to psychiatric disorders are also under stress from family conflict, including insecure attachment styles, medication compliance, and treatment programs (71, 72). Further, patients are also at risk for self-harm, victimization, and suicidal ideation (73, 74). Closer to indoor dwellings, polydrug use, inadequate alertness, suboptimal diets, bacterial and viral titers, environmental sound levels, lack of exercise or sedentary behaviors, and unemployment could all synergistically contribute to background rates of anxiety and emotional disturbances (75–79). Thus, a diverse range of environmental risk factors, including VOCs, can simultaneously affect brain networks and hormone levels (e.g., cortisol and thyroid, T4) to subsequently generate clinical symptoms of major depressive disorder and bipolar disorder (80).

Being indoors for long stretches of time not only increases the risk of exposure to harmful particle pollution (**Table 2**) but also presents unprecedented challenges to social health markers. Social health markers include marital status or cohabitation with a partner, social support groups, contact frequency with social groups, and size of the social network. It is thought that social health markers are crucial for emotional cognitive development and function at multiple scales, from individual cells to human populations (81). Not surprisingly then, deficits in social health markers such as social isolation and loneliness are risk factors for emotional cognitive dysfunction, with adolescents being particularly vulnerable, as increasing marginalization may fracture their physiological development, leading to signs of anxiety, stress, and other externalizing emotional behaviors (82, 83). Unfortunately, the specific biological mechanisms that connect bouts of social isolation to cognitive dysfunction remain unclear. However, evidence is beginning to emerge that highlights the involvement of specific neural networks of the amygdala and hippocampus and neuroendocrine markers of anxiety and stress, including cortisol levels, as potentially biological measures of social isolation and loneliness. In addition, there is data pointing to the immune system as a critical underlying factor in the trajectory of social isolation, with involvement of cytokines (e.g., interleukin-6) in the development of chronic inflammatory reactions in the brain (84–86). Note that the aforementioned neurobiological systems share key properties with signaling-secreting pathways affected by the insidiousness of airborne particles, thus raising the possibility that fractures in social health markers might be enhanced further by the simultaneous exposure to VOCs.

**Table 2:** Indoor airborne pollutants and VOCs are thought to negatively impact brain circuits specialized in emotional and cognitive function.

Volatile Organic Compounds	Sources and Hazards	References
Plasticizers	Flooring; Endocrine Risks	(101)
Benzyl Alcohol	Cosmetics; Convulsions	(102)
Methyl Chloride	Refrigerants; Drowsiness	(103)
Fire Retardants	Fire Extinguishers; Dizziness	(104)
Trichloroethylene	Cleaning Solvents; Paints; Varnishes; Headaches; Mood Swings	(105)
Stryene	Building Insulation; Drowsiness; Cognitive Impairment	(106)
Carbon tetrachloride	Cleaning Products; Refrigerants; Dizziness	(107)

Below is a list of some VOCs known to affect the human nervous system. Long-term exposure to these hazardous chemicals may influence the development or progression of mood disorders. Controlling their sources or limiting their emissions is a key strategy for improving indoor air quality.

Note that the above VOCs can cause untoward neurological and endocrine conditions and may also have a priming effect on an individual’s susceptibility to psychiatric disorders. Anthropogenic VOCs are released as mixtures of gases from soils or liquids. Due to their lipophilicity, low water solubility and volatile properties, VOCs can accumulate at elevated concentrations (> 15 mg/m^3^) in homes and buildings.

Although indoor airborne pollutants have now partly been implicated in the onset or progression of mood disorders, a comparison between noxious pollutants and known environmental stressors is warranted in order to identify phenomenological similarities as a rationale for understanding how PM_2.5_ exposure and environmental stressors acting together impact neuro-oxidative and neuro-immune signaling pathways. As mentioned earlier, major depressive disorder and bipolar disorder are chronic, generally episodic, and debilitating conditions often driven by predisposing variables such as childhood adversity, interpersonal stress, social isolation, and infection by circulating microbes (87). These environmental stressors are known to induce changes in brain monoamine-, glutamate- and GABA-signaling that in combination with elevated adrenal glucocorticoids and inflammatory cytokines contributes to the development, maintenance, and recurrence of mood disorders (88). Central to this neural, hormonal, and cell immunity response is the thesis that environmental stressors are mediated by epigenetic modifications such as DNA methylation, histone and chromatin remodeling, and non-coding RNA species that contribute to the pathological trait variability in psychiatric disorders (89, 90). Of interest, the aforementioned neural, hormonal, and cell immunity signaling can also be activated by exposure to high levels of indoor PAHs, CO, and NO, often leading to an increasingly depressive and bipolar state of anhedonia, mania, and hypomania, respectively. Thus, there is a significant overlap of multilevel biological responses that link environmental stressors with indoor VOCs. Insights from this shared link may shed light on several important queries, such as: How do VOCs exacerbate mood disorders? Are VOCs involved in the recurrence of mood disorders? And can VOCs be considered coexisting or comorbid risk elements influencing symptoms, diagnosis, and/or treatment outcomes in psychiatric disorders?

## Appropriate measures for reducing the adverse effects of indoor airborne pollutants on brain-signaling pathways

Understanding how PM_25_ exacerbates the symptoms of major depressive disorder and bipolar disorder is crucial for developing therapeutics to protect high-risk patients. A confluence of medical advances in epidemiology, imaging techniques, and *in vitro* sciences is laying the foundation for a new understanding of how indoor airborne pollution can impact biological systems, from the cellular to organismal level, to augment the development or progression of psychiatric disorders. However, it should be noted that the study of indoor airborne pollution is challenging because of differences in air exposure measuring methodology, frequency, and intensity of indoor air exposure, particularly when comparing urban, suburban, and rural communities. In addition, assessing the impact of indoor air pollution on mood disorders, both within and between patients, can be fraught with nonlinear clinical measurements as evaluation metrics used to extract biological information varied considerably, potentially introducing spurious findings and skewing the reliability of results. Moreover, a more daunting challenge is the scale and complexity of mood disorders, which has impeded rigorous molecular, neuro-anatomical, and behavioral studies for gauging the precise mechanisms underlying their distinct yet interlinked roles in brain health. Regardless of the statistical and comparative differences in measuring VOCs as well as the intrinsic properties of neurons driving mood disorders, here we provide a rather limited picture of the measures that could be taken to modify, or at least alleviate, the untoward effects of indoor air pollution on brain-signaling pathways (91–96).

- Improved air quality through exhaust ventilation, including high-efficiency particulate air filters.
- Laser light dispersion PM_2.5_ sensors to continuously monitor indoor air quality.
- Use of potted-plant systems to remove VOCs from indoor air. This strategy is to integrate existing and novel phytoremediation technology into a broader system of botanical bio-infiltration designs.
- Biological-based purification systems based on bio-catalytic action of bacteria, fungi and microalgae to improve air quality throughout homes and buildings.
- Implement emerging technologies such as artificial intelligence (AI) for measuring indoor air quality levels of CO, nitrogen oxide (NO), nitrogen dioxide (NO_2_) and sulfur dioxide (SO_2_).

## Conclusion

Here we have provided a brief summary of the environmental landscapes, biological mechanisms, and untoward consequences of indoor airborne pollution on discrete neural circuits associated with psychiatric disorders such as major depressive disorder and bipolar disorder. We must closely examine and integrate both environmental and biological evidence and examine points of agreement and contention to gain new insights into the precise contribution of VOCs on neurotransmitter anomalies thought to generate obtrusive clinical symptoms of anhedonia, mania, and hypomania.
